# Molecular mechanisms and clinical applications of gut microbiota-derived bioactive compounds in metabolic dysfunction-associated fatty liver disease

**DOI:** 10.3389/fimmu.2025.1682755

**Published:** 2025-10-20

**Authors:** Chengyun Ma, Jing Wang, Xuanli Song, Xue Wang, Shuai Zong

**Affiliations:** ^1^ Department of Clinical Laboratory, Shandong Provincial Hospital Affiliated to Shandong First Medical University, Jinan, China; ^2^ Institute for Bacterial Diseases, Jinan Center for Disease Control and Prevention Institute for Bacterial Diseases, Jinan, China; ^3^ Emergency Department, Qilu Hospital of Shandong University, Jinan, China

**Keywords:** metabolic dysfunction-associated fatty liver disease, gut microbiota-derived bioactive compound, microbiota-host crosstalk, pathogenesis, clinical application

## Abstract

Metabolic (dysfunction)-associated fatty liver disease (MAFLD) has emerged as a leading cause of chronic liver disease worldwide. Its pathogenesis is closely associated with gut microbiota dysbiosis and metabolic disturbances. In recent years, numerous studies have demonstrated that bioactive compounds produced by gut microbial metabolism—such as short-chain fatty acids, secondary bile acids, tryptophan derivatives, and bacterial extracellular vesicles—play critical roles in the development and progression of MAFLD by modulating hepatic lipid metabolism, inflammatory responses, and epigenetic regulation. The characteristic expression patterns of these gut microbiota-derived bioactive compounds provide novel options for differential diagnosis of the disease. Moreover, elucidation of the underlying pathological mechanisms has paved novel avenues for MAFLD treatment. Strategies including dietary interventions, prebiotics, probiotics, and other microbiota-targeted therapies are considered potential approaches to modulate MAFLD progression. This review systematically summarizes the molecular mechanisms underlying the development of MAFLD influenced by gut microbiota-derived bioactive compounds. It also explores the feasibility of utilizing specific gut microbial metabolite profiles for MAFLD diagnosis and highlights potential therapeutic strategies targeting microbiota-host metabolic interactions, including the use of engineered bacteria to produce specific metabolites, probiotic/prebiotic interventions, and the clinical prospects of fecal microbiota transplantation.

## 1 Introduction

Metabolic Dysfunction-Associated Fatty Liver Disease (MAFLD) is a metabolic disorder characterized primarily by hepatic fat accumulation resulting from metabolic dysfunction (1). It has become the most prevalent chronic liver disease globally (2). With the widespread adoption of nutrient-rich dietary patterns, the global burden of MAFLD continues to rise, with its reported prevalence increasing from 21.9% in 1990 to 32.4% in 2021 (3, 4). Current diagnostic methods for MAFLD include non-invasive assessments and liver biopsy. Among the non-invasive methods, imaging-based detection of hepatic steatosis is common, whereas the abdominal ultrasound alone has a sensitivity of only 20%. Controlled attenuation parameter (CAP) derived from vibration-controlled transient elastography (VCTE) and liver stiffness measurement (LSM) is primarily used for qualitative assessment, while its correlation with disease severity is limited. Although the magnetic resonance imaging (MRI) remains the gold standard for quantifying hepatic fat, it is not ideal for screening due to its high cost, time consumption, and limited accessibility. Ultrasound-guided liver biopsy, though useful for confirming atypical cases, is limited by sampling errors, cost, and potential complications (5, 6). Beyond the diagnostic challenges, therapeutic options for MAFLD remain limited. The first-line treatment still relies on lifestyle modifications such as dietary control, weight management, and physical activity. Although several drugs targeting hepatic fibrosis and inflammation have entered clinical trials, none have been approved for routine clinical use (7).

The gut microbiota (GM), a complex community of microorganisms—including bacteria, archaea, fungi, and viruses—that colonize the human gastrointestinal tract, plays a key role in host nutrient metabolism, intestinal barrier maintenance, immune system development, and metabolic homeostasis (8). In various metabolic pathways, the gut microbiota ferments dietary fibers and polysaccharides indigestible by the host, producing short-chain fatty acids (SCFAs), such as acetate, propionate, and butyrate (9). These metabolites serve as major energy sources for intestinal epithelial cells and regulate immune responses and energy metabolism. Dysbiosis, commonly observed in MAFLD patients, is marked by reduced microbial diversity and imbalanced composition, which in turn alters the types and quantities of gut microbiota-derived bioactive compounds and contributes to MAFLD pathogenesis (10).

In recent years, therapeutic agents and strategies for MAFLD targeting gut microbiota-derived bioactive compounds have emerged. However, existing reviews predominantly focus on broader microbiota compositional changes or generic “microbiota-targeted” therapeutics (11, 12), do not adequately cover the diagnostic/therapeutic potential of defined microbial metabolites. Accordingly, this review aims to synthesize current knowledge by elucidating the pathogenic mechanisms of gut microbiota-derived bioactive compounds in MAFLD, summarizing their diagnostic applications, and exploring targeted therapeutic strategies, thereby providing a comprehensive perspective toward clinical translation.

## 2 Major bioactive compounds derived from gut microbiota

### 2.1 Short-chain fatty acids

#### 2.1.1 Metabolism, functions, and targets of short-chain fatty acids

SCFAs are primarily produced via the fermentation of dietary fibers by anaerobic bacteria in the colon. These bacteria first break down dietary fiber into monosaccharides, which are then metabolized into acetate, propionate, as well as butyrate through various fermentation pathways. In addition to dietary fibers, host-derived mucins and certain amino acids and organic acids can also serve as substrates for SCFA production (13, 14). Acetate is produced by most gut bacteria, whereas propionate and butyrate are synthesized via species- and pathway-specific mechanisms—propionate mainly by Bacteroides via the succinate pathway, and butyrate by various bacteria within the Firmicutes phylum. SCFAs play central roles in energy metabolism and immune regulations (15). In host tissues, SCFAs can be converted into acetyl-CoA (based on acetate and β-oxidized butyrate) or succinyl-CoA (from propionate) to enter the tricarboxylic acid cycle (TCA) for energy production, or used for lipogenesis and gluconeogenesis (16). Each SCFA has its distinct biological roles (**Table 1**). Acetate primarily participates in energy metabolism and circulates through the portal vein, ultimately utilized by the liver and peripheral tissues. In the liver, acetate can support gluconeogenesis or fatty acid synthesis (17). At optimal concentrations, acetate also activates G-protein-coupled receptors (GPCRs), particularly GPR43, on immune cells in the portal system. Propionate is mainly metabolized in the liver to activate GPCRs, such as GPR41 and GPR43,and exert immune-regulatory functions (18). Butyrate is mainly metabolized in the colon and serves as the preferred energy source for colonocytes. Although butyrate can enter the portal circulation, its concentration in the portal vein (approximately 0.29 mM in pigs) is significantly lower than that of acetate (3.8 mM) and propionate (1.1 mM) (19). Butyrate also acts as a histone deacetylase (HDAC) inhibitor, promoting tight junction formation and mucin secretion to maintain gut barrier integrity and prevent translocation of toxins and microbial metabolites (20, 21). It further activates the AMPK pathway (22), enhancing insulin sensitivity in both hepatocytes and adipocytes, improving glucose metabolism, and reducing hepatic lipid accumulation (23). Additionally, butyrate increases the levels of anti-inflammatory cytokines (e.g., IL-10) (24) and suppresses pro-inflammatory mediators (e.g., TNF-α and IL-6) (25).

**Table 1:** Key characteristics of representative short-chain fatty acids.

Molecular formula	Representative genus	Proportion	Absorption site	Metabolic fate	Energy contribution	Ref.
Acetic acid (CH_3_COOH)	*Bacteroides, Prevotella, Ruminococcus*	60–70%	Entire colon	Liver/muscle	Systemic energy substrate	(26–29)
Propionic acid (CH_3_CH_2_COOH)	*Bacteroides, Bifidobacterium*	15–20%	Mainly proximal colon	Liver	Substrate for hepatic gluconeogenesis	
Butyric acid (CH_3_(CH_2_)_2_COOH)	*Eubacterium, Ruminococcus, Faecalibacterium, Roseburia*	10–15%	Mainly distal colon	Mainly colonic cells	Core energy source for colonic cells	

SCFAs primarily exert their effects via GPCRs that regulate physiological functions, such as hormone secretion, glucose and lipid metabolism, and immune responses (30). GPR43 has high affinity for acetate and propionate, while GPR41 has high affinity for propionate and butyrate (18). Recent studies have shown that SCFAs can reduce levels of gut pH, activating the anti-inflammatory Gαs-coupled receptor GPR65 on intestinal epithelial and immune cells (31), while other receptors, including the butyrate-specific GPR109A (32) and OLFR78 (33), are activated by acetate and propionate. However, SCFA-based treatment approaches should consider the dose effect. Some reports indicate a positive correlation between pro-inflammatory biomarkers and butyrate and propionate. Therefore, more research is needed to elucidate the mechanisms and dose effects before SCFAs supplementation is widely approved for MAFLD (34).

#### 2.1.2 Short-chain fatty acids involved in MAFLD

In MAFLD, disruptions in microbial metabolic functions lead to significant changes in the levels, proportions, and distributions of SCFAs (35). The total quantity of SCFAs is reduced due to insufficient fiber intake and microbial dysbiosis (36, 37). Particularly, the levels of protective SCFAs—propionate and butyrate—are markedly decreased, as evidenced in portal vein, peripheral blood, and fecal samples (38). The reduction in butyrate is especially critical, as it compromises energy supply to colonocytes, damages the intestinal barrier (i.e., “leaky gut”), and facilitates translocation of endotoxins (e.g., lipopolysaccharides), aggravating hepatic inflammation and accelerating the progression from simple steatosis to nonalcoholic steatohepatitis (NASH) and fibrosis (39). Furthermore, lower butyrate levels weaken the AMPK/PPARα signaling, reducing fatty acid oxidation and insulin sensitivity, thereby exacerbating gluconeogenesis and lipogenesis (40–43). Importantly, hepatic steatosis, inflammation, and insulin resistance in MAFLD further hinder SCFA transport and utilization, forming a positive feedback loop. Numerous studies have confirmed that restoring the levels of total SCFAs—especially propionate and butyrate—can exert therapeutic effects on the treatment of MAFLD, underscoring their upstream regulatory roles (**Table 2**).

**Table 2:** Recent treatments of MAFLD through the regulation of SCFAs (last 3 years).

Operation	Mechanism	Pathway	Phenotype	Ref.
Noni fruit phenolic-rich extract	Increased SCFAs level	FXR-FGF15	Reduced conversion of cholesterol to bile acids	(46)
Electroacupuncture	Increased SCFAs level	PPAR	Decreased total cholesterol and triglyceride level	(47)
Exogenous supplementation	Acetate and propionate	FFAR/AMPK	Decreased glucose, triglycerides and cholesterol	(48)
Lactoferrin	Upregulated SCFAs level	HTR2A-PPARα-CPT-1A	Reduced fatty acid synthesis and increased hepatic lipolysis	(49)
Polygonatum sibiricum polysaccharides	Increased relative abundance of Bacteroidetes	–	Decreased fasting blood glucose and fasting insulin level, improved glucose tolerance, insulin resistance, lipid and inflammatory factor level	(50)
Polysaccharides from Caulerpa lentillifera	Increased production of SCFAs (acetate and butyrate)	Bile acid axis	Improved body weight, lipid profiles and liver function	(51)
Exercise training and fiber-rich diet	Increased SCFAs level	–	Reduced fat mass and improved glucose and lipid homeostasis	(52)
Quinoa β-glucan	Increased SCFAs level	–	Significantly decreased fasting blood glucose, insulin level, triglycerides (TG) and total cholesterol (TC), while increased high-density lipoprotein cholesterol (HDLC) level; decreased malondialdehyde (MDA), aspartate transaminase (AST) and alanine transaminase (ALT) level	(53)
Sweet potato extract	Increased SCFAs level	Bile-sphingolipid metabolism	Reduced weight gain, serum low-density lipoprotein cholesterol, hepatic lipid accumulation and adipocyte hypertrophy	(54)
Resistant starch	Increased number of beneficial bacteria and SCFAs content	–	Attenuated hepatocellular vacuolation and significantly reduced number of hepatic lipid droplets	(55)
Hawthorn fruit	Increased intestinal SCFAs level	–	Inhibited weight gain and hepatic fat accumulation in NAFLD mice	(56)
Beer	Increased SCFAs level	Reduced methylation, affecting genes related to lipid accumulation and inflammation	Improved through blood parameters, weight gain, hepatic lipid content elevation and histology	(57)
Fermented barley dietary fiber	Increased SCFAs level	–	Reduced body weight and fat accumulation in the liver and epididymal white adipose tissue	(58)
Lactobacillus brevis M-10 isolated from spontaneously fermented sour porridge	Increased SCFAs content	–	Significantly reduced food intake, inhibited weight gain; prevented excessive liver growth; and decreased serum total cholesterol, triglycerides, and low-density lipoprotein	(59)
Lingguizhugan decoction	Butyrate	–	Reduced body weight, TC, TG, LDL and HDL level, significantly alleviated hepatic steatosis	(60)
Postbiotics of Lactobacillus plantarum prepared by pasteurization combined with ultrasound technology	Acetate, propionate and butyrate	SCFAs-GPR41/GPR43	Inhibited cellular triglyceride accumulation	(61)
Arctigenin	Promoted SCFA-producing bacteria and SCFA level	GPR/HDAC3, TLR4/NF-κB	Attenuated intestinal barrier damage, inflammation and Th17/Treg immune imbalance in HFD mice	(62)
Pleurotus ostreatus fermented by Lactobacillus rhamnosus	Promoted proliferation of SCFA producers	MAPK	Improved hepatic lipid accumulation	(63)
Lactobacillus plantarum NCHBL-004	Increased acetate and propionate	–	Significantly reduced weight gain, improved glucose metabolism, and maintained balanced lipid level	(64)
Exogenous supplementation of acetate, propionate and butyrate	Increased SCFAs level	GPR-HDAC3/LPS-TLR4/NF-κB/AMPK	Alleviated glycolipid metabolism disorder and liver injury	(65)
β-glucan secreted by Rhizobium pusense	Increased SCFAs level	AMPK	Reduced serum level of TC and low-density lipoprotein cholesterol (LDL-C)	(66)
Sodium butyrate	Increased butyrate level	AMPK/Sirt1/PGC-1α	Enhanced mitochondrial function and regulated systemic energy metabolism	(67)
Capsaicin	Enhanced fecal SCFAs production	PGC-1α	Significantly reversed HFF-induced obesity, improved glucose tolerance, reduced blood lipids and alleviated inflammation	(68)
Gymnemic acid	Increased relative abundance of SCFA-producing microorganisms such as Lactobacillus in the intestine	TLR4-NF-κB	Reduced inflammatory cytokines, increased expression level of antioxidant genes such as Nfe2l2, Ho-1 and Nqo1, and increased intestinal tight junction protein expression	(69)
Lactobacillus delbrueckii	Increased abundance of intestinal Lactobacillus and colonic SCFA-producing bacteria	–	Reduced lipid deposition in the liver and decreased blood lipid level	(70)
Feruloyl acetone	Increased short-chain fatty acid level	Adiponectin/AMPK/SIRT1, AMPK/PGC-1α	Prevented weight gain and enlargement of the liver and various adipose tissues	(71)
Probiotic mixture Prohep	Increased fecal SCFAs level	–	Improved hepatic steatosis, inflammation and fibrosis	(72)
Hedan tablet	Increased short-chain fatty acid level	–	–	(73)
Multi-strain probiotic WHHPRO™	Increased SCFAs level	FXR-FGF15	Improved glucose tolerance, blood lipids, body weight and liver index	(74)
Hulless barley β-glucan	Increased abundance of SCFA-producing bacteria (Prevotella-9, Bacteroides and Roseburia) and SCFA content	AMP-AMPK	Reduced hepatic lipid deposition	(75)
Jatobá-do-cerrado (Hymenaea stigonocarpa Mart.) pulp	Increased acetate and propionate or butyrate	–	Decreased triglycerides, total cholesterol, LDL-c, non-HDL-c serum level, hepatic lipids and liver weight	(76)
Dendrobium officinale Kimura & Migo	Increased SCFAs level	PPAR	Hypoglycemic effect, alleviated hepatic steatosis and impaired lipid homeostasis	(77)
ACT001	Increased valproic acid	AMPK/GPR43	Improved inflammation and intestinal mucosal barrier, reduced lipid deposition	(78)
Lactobacillus fermentum CKCC1858	Enhanced abundance of SCFA-producing bacteria	–	Decreased serum lipid level and liver function markers	(79)
Rotundic acid	Increased SCFA-producing bacteria such as Bacteroides, Anaerotruncus, Desulfovibrio, etc., significantly increased relative abundance of SCFAs	–	Reduced body weight and steatosis markers in serum and liver	(80)
Anthocyanin-Rich Butterfly Pea Flower Extract	Promoted SCFA-producing gut microbiota	–	Decreased plasma glucose, lipopolysaccharide and tumor necrosis factor-α level, restored lipid metabolism and balance between Treg and Th17 cells, inhibited dysfunctional liver and abdominal white adipose tissue	(81)
Erchen Decoction	Higher abundance of SCFA-producing bacteria	Histone deacetylase 1 and H3K9ac	Inhibited lipid metabolism disorder and reduced hepatic steatosis	(82)
Bile acids	Increased SCFAs level	FXR-PPARα	Enhanced intestinal mucosal barrier, improved intestinal morphology and altered cecal microbiota structure	(83)
Bamboo Shoots	Increased SCFAs level and SCFA-producing bacteria	–	Alleviated weight gain and liver injury in obese mice	(84)
Pomegranate peel polyphenols	Increased SCFA level and SCFA-producing bacteria	–	Reduced body weight, blood lipid and hepatic lipid level	(85)
Self-assembling polymer-based short chain fatty acid prodrugs	Increased propionate or butyrate	PPAR	Reduced hepatic lipogenesis and fibrosis	(86)
Eucommia Bark/Leaf Extract	Increased relative abundance of Ruminococcaceae. They also promoted SCFAs production	GPR41/GPR43	Alleviated lipid metabolism disorder	(87)
Polysaccharide-rich fractions from Enteromorpha prolifera	Increased SCFA-producing bacteria and intestinal barrier-protective Akkermansia muciniphila	–	Reduced HFD-induced obesity and hepatic steatosis	(88)
Prebiotic Tolypocladium sinensens	Increased SCFAs level	–	Alleviated obesity-induced inflammatory response and oxidative stress level	(89)
Prebiotic Cordyceps guangdongensis	Promoted SCFAs production	–	Reduced body weight and fat accumulation in HFD mice, improved glucose tolerance and blood lipid level, decreased lipid droplet accumulation and fat vacuole level in the liver	(90)
Prebiotic Lactobacillus delbrueckii	Increased butyrate	–	Decreased TG level, enhanced lipolysis and fatty acid β-oxidation	(91)
Lactobacillus paracasei N1115	Increased SCFAs level	–	Reduced visceral fat, liver weight, serum insulin and leptin level, and IR, and alleviated abnormal lipid metabolism	(92)
Noni fruit	Increased SCFAs production	–	Reduced body weight, decreased lipid accumulation in the liver and adipose tissue	(93)
Phycobiliproteins Bioactive Peptides	Enhanced abundance of SCFA-producing bacteria	–	Alleviated obesity by reducing body weight and improved glucose and lipid indices in serum	(94)
Sulforaphane	Increased content of Bacteroidaceae, Lactobacillaceae and Bifidobacteriaceae	GPR41/43-GLP1	Reduced blood glucose and HOMA-IRI in HFD rats	(95)

“–” indicates that the study did not report this information.

Given the well-established positive feedback effect of SCFAs in MAFLD, targeting SCFAs for therapeutic intervention has become an attractive strategy, with the potential to serve as an ideal causal treatment by modulating SCFA levels. Although numerous preclinical studies have demonstrated the benefits of SCFA supplementation, clinical trials primarily aimed at increasing SCFAs remain limited and have yet to yield strongly positive endpoint conclusions. Several ongoing clinical strategies focus on restoring SCFA levels through dietary fiber interventions or prebiotic/probiotic supplementation. A clinical trial investigating cellulose supplementation has not reported results (NCT04520724). Similarly, two clinical trials evaluating SCFA-producing probiotics have not yet published outcomes (NCT06491342, NCT05402449) (44). Another study on a synbiotic formulation designed to boost SCFAs is still ongoing (NCT05821010). Therefore, the clinical value of SCFAs requires further validation through more clinical studies rather than additional preclinical research. Important future directions include developing precise delivery systems for SCFAs or their analogs to enhance colonic bioavailability, and defining optimal SCFA intervention strategies for different stages of MAFLD. Since SCFA levels can be influenced by multiple metabolic factors, constructing composite diagnostic models that incorporate SCFAs represents a feasible approach for using SCFAs as non-invasive biomarkers for MAFLD. For instance, Lin et al. developed an integrated model including physical indicators, laboratory parameters, and gut metabolite SCFAs, which demonstrated strong diagnostic performance (AUC=0.938) (45). However, the clinical applicability of such models depends on validation in multicenter studies and their ability to differentiate MAFLD from other conditions.

### 2.2 Bile acids

#### 2.2.1 Metabolism, functions, and targets of bile acids

Primary bile acids (BAs) are synthesized in the liver and metabolized by gut microbiota (primarily including *Bacteroides, Clostridium, Lactobacillus, Bifidobacterium*, etc.) into secondary BAs (96). The ratio of primary to secondary BAs plays a key role in regulating lipid absorption efficiency and metabolic signaling transduction (97, 98). As signaling molecules, both primary and secondary BAs can act as ligands for the farnesoid X receptor (FXR) (99). Upon activation of intestinal FXR, it stimulates enteroendocrine cells to secrete fibroblast growth factor 15/19 (FGF15/19) (100). Once the hormone reaches the liver, it binds to specific receptors on the liver cell membrane (a complex formed by FGFR4 and the co-receptor β-Klotho), which subsequently inhibits the BA synthesis enzyme CYP7A1 (101, 102). This creates a negative feedback loop regulating the size of the BA pool, while simultaneously improving glucose and lipid metabolism, i.e., inhibiting lipogenesis, promoting fatty acid oxidation, and enhancing insulin sensitivity (99). Additionally, BAs can activate the G protein-coupled bile acid receptor 1 (TGR5), exerting effects on enteroendocrine cells, macrophages, hepatocytes (103), and other cell types, for example, promoting GLP-1 release to improve glucose metabolism and satiety and inhibiting macrophage inflammatory responses to reduce inflammation (104, 105).

BAs also possess antimicrobial activity. Changes in their composition and concentration directly impact the structure of the gut microbiota (106). Conversely, dysregulation of BA metabolism can lead to dysbiosis, e.g., an increase in the relative proportion of lipopolysaccharide (LPS)-producing bacteria, forming an interacting cycle (107).

#### 2.2.2 Bile acid metabolism in MAFLD

BA metabolism is under tight control through a complex feedback loop involving the liver, gut, and gut microbiota. In MAFLD patients, abnormalities often occur in BA synthesis, metabolism, and signaling (108, 109). Specifically, signaling through the receptors FXR and TGR5 is frequently reduced, which links to problems like insulin resistance, fat accumulation in the liver (steatosis), and inflammation (98).

Studies have shown that the levels and types of BAs are altered in MAFLD patients, detected in their liver, blood, and stool (110). Typically, in the blood, the levels of primary BAs, such as cholic acid (CA) and chenodeoxycholic acid (CDCA), are increased. A key enzyme responsible for making 12α-hydroxylated (12-OH) BAs, called CYP8B1, is also activiated (111). This leads to a significant increasd in the levels of 12-OH BAs like CA and deoxycholic acid (DCA). Importantly, the ratio of these 12-OH BAs to non-12-OH BAs serves as a key marker for metabolic performance in MAFLD, correlating strongly with the levels of liver fat and inflammation (108, 112). Conversely, the levels of non-12-OH BAs, such as ursodeoxycholic acid (UDCA) and lithocholic acid (LCA), tend to be lower in MAFLD patients. Since these acids often have protective anti-inflammatory effects, the reduction in the levels of these acids could worsen the disease (98). Excretion of secondary bile acids like DCA and LCA is also reduced. This happens because imbalances in gut bacteria impair the conversion of primary to secondary BAs. Consequently, this limits the ability of these secondary acids to activate beneficial receptors like FXR and TGR5, which normally regulate fat metabolism and reduce inflammation. Therefore, finding ways to restore a healthy balance (homeostasis) in BA metabolism is considered a promising potential treatment for MAFLD (113).

Therapeutic strategies targeting BAs for MAFLD warrant extensive exploration. Bile acid signaling pathways, particularly those involving FXR and TGR5, are emerging as key pharmacological targets for metabolic diseases. Obeticholic acid (6α-ethyl-chenodeoxycholic acid, an FXR agonist) has been shown to improve histological features of NASH and demonstrated favorable outcomes in a Phase III clinical trial (NCT01265498) (114–116). Two novel FXR agonists, cilofexor and TQA352, have successfully passed clinical safety assessments (NCT02781584, ChiCTR1800019570) (117). The development of TGR5 agonists aims to leverage their ability to promote GLP-1 secretion and suppress inflammation, thereby improving glycemic control and mitigating hepatic inflammation. One clinical study observed increased TGR5 expression levels in peripheral blood mononuclear cells and reduced liver fat content in patients following curcumin supplementation (ChiCTR2200058052) (118).

It is important to note that targeting these pathways requires precise balance. For instance, obeticholic acid has been associated with side effects such as an increased risk of drug-induced liver injury, unfavorable changes in lipid levels, and severe pruritus (119–121), leading the FDA to deny its conditional approval for NASH. This underscores the importance of developing tissue-specific agonists/antagonists. Beyond directly targeting signaling pathways, other BA-focused therapeutic strategies should be considered. For example, modifying the bile acid pool composition using non-12-OH bile acids like UDCA and its derivatives has shown promise. An 18-week treatment with berberine ursodeoxycholate resulted in histological improvement in most MAFLD patients, with dose-dependent improvements observed across various biomarkers (NCT03656744) (122). Furthermore, a clinical study demonstrated that aerobic exercise increased total bile acid and ursodeoxycholic acid levels in MAFLD patients, significantly improving body composition and liver function while also reducing blood lipid and glucose levels (NCT06338449). Additionally, microbiome intervention strategies—using probiotics or prebiotics to restore microbial function and promote the production of secondary bile acids (e.g., LCA)—should be explored to achieve natural and mild activation of the FXR/TGR5 pathways. On the diagnostic front, analyzing the bile acid pool represents a potential non-invasive strategy, but it should be combined with other diagnostic approaches to avoid confounding factors from gallbladder diseases.

### 2.3 Tryptophan derivatives

#### 2.3.1 Metabolism, functions, and targets of tryptophan derivatives

Gut microbiota uses tryptophan as a precursor to generate various derivatives through specific enzyme systems (123). Bacteria like *Clostridium*, *Bacteroides*, and *Bifidobacterium* are involved, producing compounds such as indole, indole-3-propionic acid (IPA), tryptamine, and indole-3-acetic acid (IAA) (124). For example, Enterobacteriaceae produce indole by deaminating tryptophan via tryptophanase (125); *Lactobacillus* sp. generates tryptamine catalyzed by aromatic amino acid decarboxylase (126); Clostridium creates IPA through hydroxylation (127); and Bacteroides synthesizes IAA (128–130). Once absorbed by the host, these metabolites undergo further processing, i.e., indole is oxidized by hepatic CYP2E1 (131) or SULT1A1 (132) into indoxyl, then sulfated or glucuronidated for excretion; IPA acts freely after sulfation; and tryptamine is degraded by host monoamine oxidase (MAO) into indoleacetic acid.

Tryptophan derivatives support gut barrier integrity, immune regulation, and metabolic control (133). Indole and IPA activate the aryl hydrocarbon receptor (AhR), boosting expression of tight junction proteins (occludin and claudin-1) and mucins (MUC2), strengthening the gut barrier (134). IAA accelerates mucosal repair by regulating intestinal stem cell proliferation. Through AhR activation, indole promotes regulatory T cell (Treg) differentiation and IL-10 secretion, curbing Th17-driven gut inflammation (127), while IPA inhibits pro-inflammatory cytokines like TNF-α and IL-6 from macrophages (135), exerting its anti-inflammatory effects by regulating AhR-NLRP3 axis (136).

#### 2.3.2 Tryptophan derivatives in MAFLD

Studies have shown an inverse link between indole levels and liver fat content—obese individuals typically have lower indole and higher hepatic fat (137). The beneficial roles of tryptophan derivatives in MAFLD are well-documented (138, 139). Indole and its derivatives exhibit anti-inflammatory properties by increasing the levels of IL-10 andinhibiting TNF-α–driven NF-κB activation and pro-inflammatory chemokine IL-8 expression. This helps maintain gut barrier functions and has been demonstrated in both cellular and animal models to reduce liver steatosis and fibrosis (140, 141). In addition, targeting the AhR pathway has been shown to inhibit lipid accumulation in the liver, reduce the level of triglycerides and total cholesterol, and alleviate oxidative stress (142). Research by Ding et al. demonstrated that exogenous administration of indole-3-acetate (I3A) improved hepatic pathology without altering the gut microbiota state, suggesting its direct effect on hepatic metabolic function (143). Indoleamine 2,3-dioxygenase (IDO), a key rate-limiting enzyme in gut tryptophan metabolism, catalyzes the conversion from tryptophan to kynurenine. Research in high-fat-diet-fed IDO-knockout (IDO^-^/^-^) mice revealed less inflammatory macrophage infiltration and reduced susceptibility to obesity-linked fatty liver and insulin resistance (144) suggesting the beneficial roles of indole and its derivatives in MAFLD.

In MAFLD, tryptophan metabolism shifts towards the detrimental kynurenine pathway, while the beneficial microbial pathways (such as the production of AhR agonists) are suppressed. This imbalance directly contributes to intestinal barrier disruption, systemic inflammation, and hepatic steatosis (145). Therefore, restoring tryptophan metabolic balance represents an etiology-targeting strategy. Potential therapeutic approaches include using specific probiotics to remodel gut microbiota function and enhance the production of endogenous beneficial metabolites, as well as exploring drugs like IDO1 inhibitors to reduce the generation of pro-inflammatory kynurenines. From a diagnostic perspective, blood levels of indole/IPA or the indole/kynurenine ratio show promise as non-invasive biomarkers for assessing gut ecological function and liver disease severity. However, further research is needed to clarify the discriminatory power of such models.

### 2.4 Trimethylamine N-oxide

Gut microbes convert precursor substances such as choline, L-carnitine, and phosphatidylcholine ingested by the host into trimethylamine (TMA). The produced TMA enters the liver via the bloodstream, where it is oxidized by flavin-containing monooxygenases (FMOs) into trimethylamine N-oxide (TMAO) (146). In recent years, multiple studies have reported the correlation between TMAO levels and the pathogenesis of MAFLD. A clinical investigation by Ma et al. found that higher blood TMAO concentrations were associated with an increased risk of MAFLD (147). Additionally, fecal TMAO levels have been shown to correlate with the severity of MAFLD (148). Subsequently, numerous experimental studies have demonstrated that TMAO disrupts lipid metabolism and promotes the occurrence and progression of MAFLD (149, 150).

The promotion of MAFLD by TMAO involves multiple phenotypes and pathways. Some studies have reported that TMAO exacerbates hepatic steatosis by inhibiting bile acid (BA)-mediated hepatic FXR signaling (151). Furthermore, TMAO exhibits pro-inflammatory properties by activating the TLR4/MyD88/NF-κB signaling pathway, upregulating the expression of various inflammation-related genes, and simultaneously inducing the polarization of liver macrophages toward the pro-inflammatory M1 phenotype, thereby triggering liver inflammation (148). Novel mechanisms of TMAO-mediated MAFLD have also been discovered. For example, TMAO inhibits OTUB1-mediated SLC7A11 stability, leading to hepatocyte ferroptosis and accelerating MAFLD progression (152). TMAO upregulates the expression of HULC, followed by P38MAPK overexpression, thereby mediating hepatocyte apoptosis and promoting MAFLD development (148). Yang et al. found that TMAO can activate the PERK signaling pathway, subsequently inducing MAFLD (153).

However, it is important to note that conflicting research conclusions exist. Miyata et al. reported that after feeding FXR-null mice a diet containing 0.3% TMAO for 13 weeks, markers of liver injury were significantly reduced, suggesting that TMAO may improve liver function through pathways independent of bile acid metabolism (154). Therefore, more studies with variables such as dosage and administration duration are needed to clarify the precise role of TMAO in MAFLD.

The aforementioned findings establish TMAO as a significant risk biomarker and potential pathogenic factor in the onset and progression of MAFLD, warranting consideration for incorporating TMAO levels into diagnostic models aimed at assessing MAFLD risk and disease severity. It should be noted that the association of TMAO with conditions such as cardiovascular disease and cancer has been extensively reported (155, 156), suggesting the potential of developing composite biomarker diagnostic models based on TMAO. On the therapeutic front, evaluating dietary interventions that reduce the intake of precursor substances rich in choline and L-carnitine becomes important. Furthermore, the development of FMO enzyme inhibitors to block the conversion of TMA to TMAO, along with preclinical and clinical trials to validate their therapeutic value, represents a viable strategy.

### 2.5 Endotoxins

Endotoxins (i.e., LPS), major components of Gram-negative bacterial cell walls in Enterobacteriaceae, strongly activate the host innate immune system (157). In MAFLD, LPS acts as a key mediator of “leaky gut”—translocating into the liver via the portal vein when the intestinal barrier is compromised (158–160). As the primary ligand for Toll-like receptor 4 (TLR4), which is expressed in hepatocytes, Kupffer cells (liver macrophages), and hepatic stellate cells, LPS binding triggers downstream pathways like MyD88-dependent signaling (161). This activates transcription factors such as NF-κB, driving production of pro-inflammatory cytokines (TNF-α, IL-1β, and IL-6) and chemokines (162)—key mechanisms regulating liver inflammation, cell damage, and progression to NASH. TLR4 signaling also induces insulin resistance by disrupting insulin receptor substrate pathways (163). Sustained inflammation activates hepatic stellate cells, promoting extracellular matrix deposition and fibrosis. Besides TLR4, LPS recognition involves LPS-binding protein (LBP) and CD14 (164). Clinically, elevated LBP levels correlate with insulin resistance and dyslipidemia in non-alcoholic fatty liver disease (NAFLD) or NASH patients (165). In high-fat-diet MAFLD models, LBP-knockout mice show improved lipid metabolism and milder pathology (166). LPS can cleave membrane-bound CD14 (mCD14), releasing presepsin into circulation (167), while CD14 depletion reduces liver lipids and macrophage content, ultimately alleviating steatosis (168). Both MAFLD patients and animal models exhibit increased serum LPS (169). Studies have demonstrated that antibiotic treatment (e.g., polymyxin B targeting Gram-negative bacteria) effectively lowers TNF production and plasma LPS levels, reversing hepatic steatosis (170).

Therapeutic strategies targeting LPS hold promising potential for clinical exploration. As a central mediator linking “leaky gut” to hepatic inflammation, LPS acts as an accelerator in the pathogenesis of MAFLD. Its multi-faceted mechanisms—driving liver inflammation, insulin resistance, and fibrosis through the TLR4 signaling pathway—make it a valuable therapeutic target. From a diagnostic perspective, the levels of serum LPS, LBP, or CD14 should be considered as potential non-invasive biomarkers for evaluating intestinal barrier function and systemic inflammatory status.

### 2.6 Bacterial extracellular vesicles

Both Gram-positive and Gram-negative bacteria produce bacterial extracellular vesicles (BEVs) (171). Under normal physiological conditions, the liver manages the physiological stress caused by BEVs. However, in MAFLD, dysbiotic gut microbiota releases excessive BEVs loaded with bioactive bacterial components (e.g., LPS, bacterial DNA, and proteins) (172). These vesicles cross the compromised gut barrier (“leaky gut”) and enter the liver via the portal circulation. BEVs from different bacteria exert harmful effects through distinct molecular activations, i.e., outer membrane vesicles (OMVs) from Gram-negative bacteria are LPS-rich, with lipid A specifically recognized by TLR4 (173); cytoplasmic membrane vesicles from Gram-positive bacteria express lipoteichoic acid, activating TLR2 (174); and BEVs from pathogens like *Porphyromonas gingivalis* (with high LPS levels) induce M1 macrophage polarization and amplify pro-inflammatory responses (175). By delivering virulence factors directly to host cells, BEVs significantly contribute to inflammation and disease progression. Fizann et al.’s research reinforces the detrimental role of BEVs in disease states, demonstrating that administration of fecal-derived extracellular vesicles (fEVs) from NASH patients upregulates pro-fibrotic and pro-inflammatory protein expression in hepatic stellate cells and increases intestinal permeability in wild-type mice. Their study specifically highlighted the pathogenic contributions of nmMLCK and LPS carried within BEV cargo (176). Critically, additional evidence implicates BEV-carried DNA in pathology: Luo et al. demonstrated that Vsig4+ macrophage deficiency in disease states facilitates translocation of microbiota-containing extracellular vesicles (mEVs), leading to accumulation of microbial DNA in hepatocytes and hematopoietic stem cells. This subsequently activates the cGAS/STING signaling pathway, mediating inflammatory responses (177).

**Figure 1:**
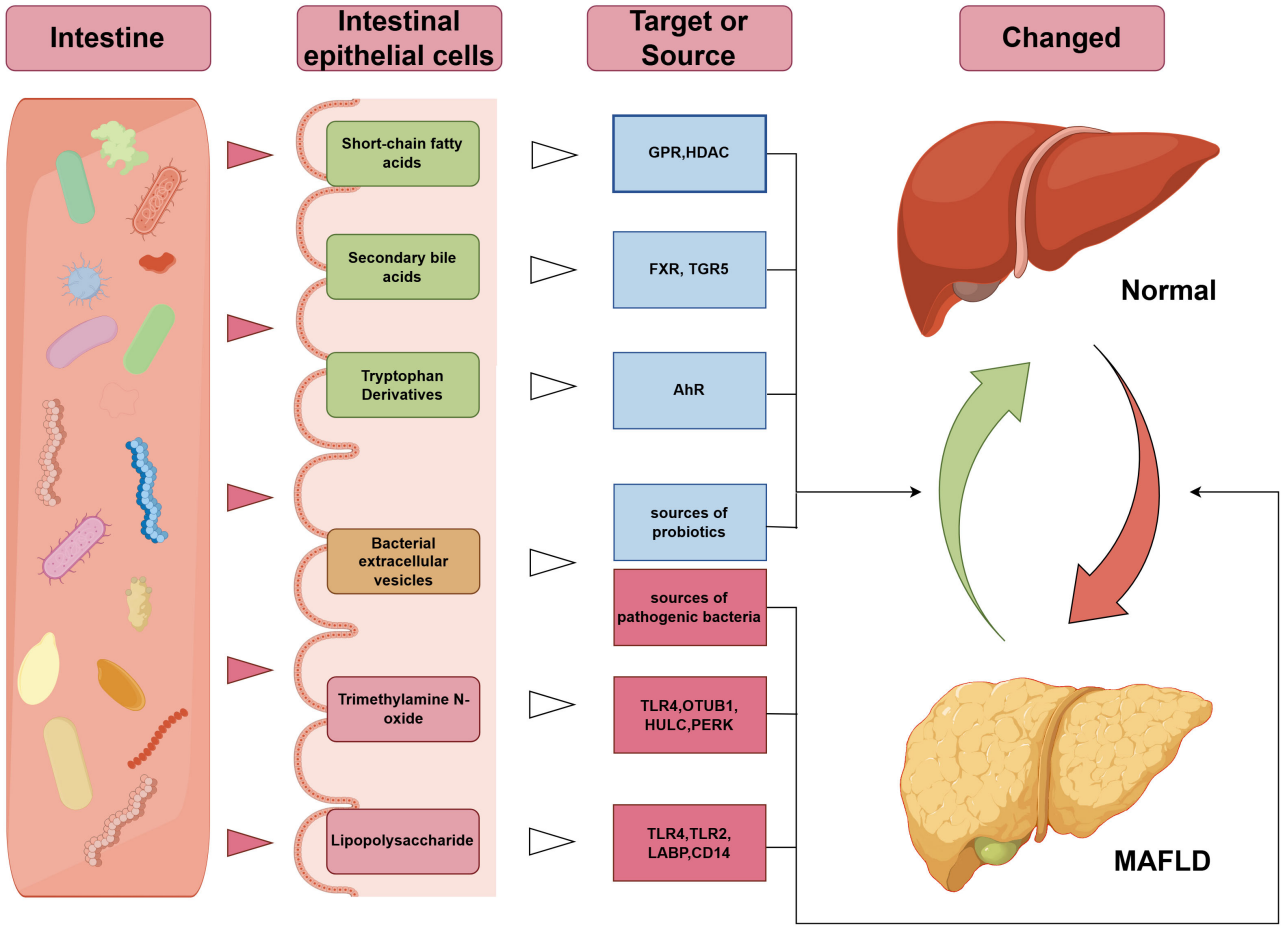
Mechanisms underlying the association between gut microbiota-related bioactive substances and MAFLD. The green terms in the “intestinal epithelial cells” column indicate beneficial components, yellow represents neutral components, and red indicates harmful components; the blue terms in the “Target or Source” column denote beneficial targets, while red denotes harmful targets. GPR, G-Protein Coupled Receptor; HDAC, Histone Deacetylase; FXR, Farnesoid X Receptor; TGR5, Takeda G-protein-coupled Receptor 5; AhR, Aryl Hydrocarbon Receptor; TLR, Toll-Like Receptor; OTUB1, OTU deubiquitinase, binary 1; HULC, Highly Upregulated in Liver Cancer; PERK, PKR-like Endoplasmic Reticulum Kinase; LBP, Lipopolysaccharide-Binding Protein; CD14, Cluster of Differentiation 14.

It should be noted that previous studies have also confirmed the beneficial effects of beneficial intestinal bacteria on disease phenotypes, for instance, BEVs from lactic acid bacteria demonstrate efficacy in reducing oxidative damage (178), and studies confirm the protective effects of Enterococcus faecium-derived EVs against ethanol-induced hepatic injury in rats (179). Therefore, the source of gut bacteria and the type of cargo content may constitute the pivotal determinant underlying the double-edged effects of BEVs. There is an urgent need for in-depth research to differentiate the roles of BEV origin and cargo composition in MAFLD (**Figure 1**).

BEVs hold broad potential for exploration, primarily encompassing applications as non-invasive diagnostic biomarkers and targeted therapeutic vehicles. For instance, specific BEVs in the blood or their cargo—such as microbial DNA and proteins—could serve as novel non-invasive biomarkers for assessing gut microbiota status and MAFLD disease activity. Additionally, engineered exosomes derived from intestinal probiotics are being investigated for their ability to precisely deliver anti-inflammatory or metabolic regulatory factors, combining favorable biocompatibility with therapeutic efficacy.

### 2.7 Crosstalk among bioactive substances

It is important to note that gut microbiota-derived bioactive substances do not exert their biological functions independently but rather regulate each other’s metabolic homeostasis through various crosstalk mechanisms, thereby achieving integrated regulation of hepatic lipid metabolism. Studies have reported the mechanisms by which SCFAs influence bile acid metabolism. Tolhurst et al. discovered that short-chain fatty acids can trigger the secretion of glucagon-like peptide-1 (GLP-1) (180). In a clinical study, GLP-1 agonists demonstrated even better efficacy in treating bile acid diarrhea than the standard-of-care bile acid sequestrant colesevelam (181), suggesting that short-chain fatty acids play a positive role in stabilizing bile acid metabolism. Lu et al. indicated that short-chain fatty acids activate the FXR-FGF15-CYP7A1 pathway, reducing bile acid synthesis and improving bile acid metabolism (182). Crosstalk between tryptophan and bile acid metabolism has also been reported. Chen et al. found through exogenous supplementation in mice that tryptophan inhibits intestinal FXR signaling and promotes hepatic bile acid synthesis and excretion, accompanied by elevated levels of conjugated bile acids and an increased ratio of non-12-OH to 12-OH bile acids in hepatic and fecal bile acid profiles (183). IDO-1 is the rate-limiting enzyme in tryptophan degradation. However, Qiao et al. demonstrated that inhibition of IDO-1 expression leads to a decrease in SCFA levels (184), suggesting a crosstalk effect between tryptophan or its derivatives and SCFAs, though further research is needed to elucidate the underlying mechanisms. In summary, crosstalk exists among gut bioactive substances such as SCFAs, bile acids, and tryptophan metabolites. Abnormal levels of any of these gut microbiota-derived bioactive compounds may impact the levels of others, ultimately leading to changes in the overall metabolic network. More research is needed to achieve a deeper understanding of this network, which could aid in the formulation of postbiotic combination therapies.

## 3 Diagnostic potential of gut microbiota-derived bioactive compounds in MAFLD

The dysregulation of the gut microbiome and its bioactive compounds is closely linked to MAFLD progression, offering a promising diagnostic tool for MAFLD (36). Advances in multi-omics technologies have made it feasible to leverage gut microbiota-derived bioactive compounds for MAFLD diagnosis (12). For instance, Zhang et al. studied 60 MAFLD patients and developed a metabolomics model centered on propionate and butyrate analogues as key differentially expressed markers. This model achieved an AUC of 0.94, outperforming phenomics (AUC=0.91) and gut metagenomics (AUC=0.78), and combined metabolomics-phenomics model further improved diagnostic accuracy (AUC=0.97) (185). Beyond distinguishing MAFLD from healthy individuals, these compounds can also identify MAFLD comorbidities. For example, Li et al. used a random forest (RF) machine learning algorithm integrating gut metagenomics and plasma metabolomics to recognize MAFLD patients at risk of cardiovascular disease (186). Furthermore, monitoring disease progression using gut microbiota-derived bioactive compounds has also been reported. Luo et al. demonstrated through targeted metabolomics that nine metabolites are involved in the metabolic reprogramming of MAFLD-related inflammation. They constructed a machine learning model using seven of these inflammation-related metabolites to assess MAFLD disease progression (187). Lin et al. established a comprehensive model incorporating short-chain fatty acid/tryptophan metabolites and clinical variables such as arm circumference, which showed good predictive power for severe liver steatosis (45). In addition to building diagnostic models, some studies have reported independent risk metabolites for MAFLD. Barrea et al. found that Trimethylamine N-oxide (TMAO) can serve as an early biomarker for adipose tissue dysfunction and NAFLD even in the absence of overt metabolic syndrome, suggesting that a TMAO-based threshold could help identify the NAFLD population (188).

BEVs deserve special attention as diagnostic tools due to their accessibility and non-invasive nature. Their stronger immunogenicity compared to host-derived vesicles enhances diagnostic specificity (189). Studies have shown significantly elevated LPS-positive BEVs in plasma from patients with gut barrier dysfunction, correlating positively with plasma ZO-1 levels, indicating reduced mucosal integrity and increased permeability. This positions LPS-positive BEVs as promising biomarkers for intestinal barrier damage (172, 190). However, diagnostic potential varies based on the sources of BEVs source, i.e., fecal BEVs are abundant but prone to environmental contamination during collection, limiting their accuracy for systemic conditions, while blood-derived BEVs reflect more accurately the whole-body status but face technical challenges in isolation and characterization due to low biomass, typically yielding less material than fecal samples (191).

Despite these encouraging findings, the current research landscape has limitations. Many studies are preliminary, with small sample sizes, and lack validation in large, independent cohorts. The diagnostic models often require further refinement for clinical application. For BEVs, standardized protocols for isolation and characterization are urgently needed. Therefore, while existing evidence robustly confirms the principle that microbial products can serve as biomarkers, future research must focus on translational validation, standardization of assays, and determining the incremental value of these biomarkers over established clinical parameters.

## 4 Therapeutic strategies targeting gut microbiota-derived bioactive compounds

Modulating levels of SCFAs, BAs, and tryptophan derivatives has proven effective therapeutic treatment against MAFLD. For instance, Yan et al. demonstrated that fecal microbiota transplantation from mice treated with *Morinda citrifolia* polyphenol extract elevated SCFA-producing bacteria. The resulting increased levels in SCFAs activated the intestinal FXR-FGF15 pathway, subsequently triggering hepatic FXR to suppress CYP7A1 expression—thereby regulating cholesterol-to-BA conversion and maintaining lipid homeostasis (46). Liu et al. found electroacupuncture (EA) improved adipose tissue pathology and reduced the levels of total cholesterol/triglycerides by modulating lipid metabolism-associated gut microbiota, increasing the levels of SCFAs, and activating PPAR signaling (47). Wu et al. showed that *Akkermansia muciniphila* supplementation reshaped BA profiles by regulating the gut FXR-FGF15 axis (192). Nie et al. identified multiple microbially modified BAs, including the previously uncharacterized 3-succinylated cholic acid (3-sucCA), that inversely correlated with liver injury in biopsy-confirmed MAFLD patients. Furthermore, 3-sucCA alleviated MASH by promoting *A. muciniphila* (193). Moreover, oral administration of indole-3-acetate has been demonstrated in preclinical studies to suppress the expression of several enzymes involved in hepatic lipogenesis and beta-oxidation, while concurrently mediating anti-inflammatory effects in macrophages through the AMPK signaling pathway (143).

Dietary interventions, probiotics/prebiotics, microbiota transplantation, engineered bacteria, and postbiotics all show therapeutic promise. However, each has its own advantages and disadvantages (**Table 3**). Dietary intervention is a relatively safe treatment strategy. High-fiber diets provide abundant SCFA precursors (194), while Mediterranean diets—rich in polyphenols, polyunsaturated fatty acids (PUFAs), oleic acid, carotenoids, and fiber—exert antioxidant, anti-inflammatory, and antimicrobial effects (195). Both types of diets are recognized as MAFLD mitigators. A meta-analysis integrating 11 studies assessing Mediterranean diet adherence scores demonstrated that this dietary strategy significantly reduced body weight and alanine aminotransferase (ALT) levels, suggesting its efficacy in supporting weight loss and improving liver health in patients with MASLD/MASH (196). Clinical observations indicate that a daily intake of 24 grams of fiber reduces hepatic steatosis and significantly lowers aspartate aminotransferase (AST) and total cholesterol levels (197). However, patient compliance, individual variability, and delayed efficacy necessitate positioning dietary therapy as a foundational approach. Meanwhile, Probiotic/prebiotic supplements also demonstrate benefits. A clinical study demonstrated that short-term probiotic supplementation can improve ALT, AST, and BMI (NCT06074094) (198). Supplementing with prebiotics alone has also yielded positive clinical results. For example, Lycium barbarum polysaccharide (LBP) supplementation reduced ALT levels in MAFLD patients (ChiCTR2000034740) (199). It should be noted that most prebiotic research remains at the preclinical stage. For instance, Ma et al. revealed that *Tricholoma mongolicum* polysaccharide (TMP) significantly enhanced gut microbial α-diversity in MAFLD models, restructured community composition, lowered Firmicutes/Bacteroidetes ratios, and enriched SCFA-producing microbial genera. Proteomics confirmed that TMP suppressed hepatic immune inflammation and ferroptosis while enhancing metabolic homeostasis pathways (200). Whether these agents have clinical translational value requires further validation. Engineered bacteria represent another group of frontiers. Zhang et al. used dual-targeted nanoparticles hitchhiking on Lactobacillus *rhamnosus* to enhance gut accumulation and liver-targeted delivery of anthocyanins, improving MAFLD treatment (201). However, it must be noted that safety concerns, bioethical concerns and industrial-scale production hurdles remain. Fecal microbiota transplantation (FMT) was previously considered a potential therapeutic approach and demonstrated success in preclinical studies (202). However, conflicting results in clinical trials have introduced uncertainty regarding this strategy. The study by Xue et al. reported that allogeneic FMT administered three times within three days reduced hepatic fat accumulation (203). However, Groenewegen et al. found no significant effects of FMT on liver steatosis, glucose tolerance, hepatic biochemistry, or gut microbiota composition in their clinical trial (NCT04465032) (204). Craven et al. reached an intermediate conclusion, demonstrating that FMT did not improve hepatic proton density fat fraction (PDFF) but might reduce intestinal permeability in patients with MAFLD (NCT02496390) (205). Moreover, FMT still faces significant challenges before it can be widely adopted in clinical practice, including infection risks, potential dysbiosis, and ethical dilemmas (206). The clarified roles of gut microbiota-derived bioactive compounds have paved the way for postbiotics. These overcome limitations of live bacteria (i.e., colonization issues, stability challenges, and dosing precision) and stand as a promising MAFLD treatment strategy (207). Clinical trials by Fogacci et al. have demonstrated that a butyrate-based therapeutic strategy reduces hepatic steatosis scores and improves key lipid profile indicators (208).

**Table 3:** Advantages and limitations of gut microbiota regulation strategies in MAFLD.

Strategy category	Representative method	Advantage	Limitation	Ref.
Dietary intervention	High-fiber diet	High safety with no side effects	Great differences in compliance	(197)
		Increase SCFAs production and improve insulin sensitivity	Slow onset (requires long-term adherence)	
		Regulate gut microbiota diversity	Significant individual response heterogeneity	
Probiotics/prebiotics	Bifidobacteria + Fructooligosaccharides (synbiotics)	Non-invasive with good tolerance	Strong strain specificity and unstable efficacy	(209)
		Enhance intestinal barrier and inhibit LPS translocation	Colonization resistance affects long-term efficacy	
		Some strains have enzyme-lowering effects	Lack of standardized preparations	
Postbiotics	Butyrate preparations	Avoid the problem of viable bacteria colonization	Immature colon-targeted delivery technology	(210)
		High stability and controllable dosage	High doses may cause gastrointestinal discomfort	
		Directly supplement active metabolites	Relatively high cost	
Fecal microbiota transplantation	Transplantation of microbiota from healthy donors	Rapidly reshape microbiota structure	Risk of infection/microbiota disorder	(211)
		Potential reversal of insulin resistance	Doubts about the durability of efficacy	
			Lack of ethics and standardization	
Engineered bacteria therapy	Dual-Targeted Nanoparticles Hitchhiking on Lactobacillus rhamnosus	Precisely degrade specific toxins	Controversies over biosafety	(201)
		Programmable control of microbiota functions	Difficulty in industrial production	
			Unknown immunogenicity	

## 5 Conclusion

MAFLD, the most prevalent chronic liver condition worldwide, is increasingly linked to gut dysbiosis and metabolic disruption. This review systematically details how gut microbiota-derived bioactive compounds—SCFAs, BAs, tryptophan derivatives, MTAO, endotoxins, and bacterial extracellular vesicles—orchestrate MAFLD progression from steatosis to steatohepatitis and fibrosis. These gut microbiota-derived bioactive compounds directly or indirectly modulate hepatic lipid metabolism, gut barrier integrity, immune responses, and signaling pathways (e.g., FXR, TLR4, and AMPK). Clinically, characteristic microbial metabolite profiles offer novel diagnostic biomarkers (e.g., propionate/butyrate ratios for SCFAs and 12-OH/non-12-OH BA ratio) to enhance diagnostic accuracy. BEVs, with their high specificity and accessibility, show promise as non-invasive indicators for MAFLD progression. Therapeutically, strategies targeting gut microbiota-host metabolic linkages—high-fiber diets, probiotics/prebiotics, FMT, engineered bacteria, and postbiotics—hold significant potential for the treatment of MAFLD. By restoring microbial balance and metabolite homeostasis (e.g., boosting the levels of SCFAs, and normalizing BAs), they effectively combat hepatic lipid accumulation and inflammation. Currently, elucidating the role of gut microbiota-related bioactive substances in MAFLD remains challenging. Firstly, while most studies suggest that SCFA supplementation is beneficial for individuals, there are also reports of ineffective or even harmful outcomes. Similarly, conflicting research results exist for BAs and TMAO, necessitating more precise variable control to clarify the effective and harmful ranges of these bioactive substances, thereby unlocking their potential for clinical applications. Secondly, the heterogeneity of individual microbiomes affects diagnostic consistency. Relatively few studies focus on the diagnostic utility of gut-derived bioactive substances, highlighting the need for larger sample sizes and stricter enrollment criteria to develop diagnostic models with clinical applicability. Additionally, the mechanisms of certain bioactive compounds (e.g., BEVs) in MAFLD progression are not yet fully understood. Greater efforts to clarify the roles of these bioactive compounds will facilitate drug target design and translation. In terms of clinical translation, challenges such as probiotic colonization efficiency, the safety of FMT, and ethical concerns regarding engineered bacteria remain significant obstacles. Further discussions are needed to determine the most suitable therapeutic strategies for MAFLD.
